# Polyarteritis nodosa complicated by posterior reversible encephalopathy syndrome: a case report

**DOI:** 10.1186/1756-0500-7-89

**Published:** 2014-02-14

**Authors:** Mitrakrishnan Rayno Navinan, Chandrika Jayakanthi Subasinghe, Thambyaiah Kandeepan, Aruna Kulatunga

**Affiliations:** 1 National Hospital of Sri Lanka, Colombo, Sri Lanka

**Keywords:** Polyarteritis nodosa, Posterior reversible encephalopathy syndrome, Vasculitis

## Abstract

**Background:**

Posterior reversible encephalopathy syndrome is a presentation which is diagnosed clinico-radiologically. The primary aetiological processes leading to posterior reversible encephalopathy syndrome are many, which include autoimmune conditions. Polyarteritis nodosa as an aetiological factor for posterior reversible encephalopathy syndrome is rare. We present a case of polyarteritis nodosa complicated by posterior reversible encephalopathy syndrome.

**Case presentation:**

A 26-year-old South-Asian female presented with left sided focal seizures with secondary generalization and visual disturbance for 2 days duration. She had a prior history of arthralgia and weight loss with no medically explainable cause for young onset hypertension. Examination revealed a right claw hand with a palpable vasculitic type of rash involving both the palmar surfaces. Symptoms responded to management with anti-hypertensives and anti-epileptics. Whole blood count, iron studies, erythrocyte sedimentation rate and C-reactive protein values portrayed an ongoing chronic inflammatory process. Serological studies such as Anti-nuclear antibody, Anti -double stranded deoxyribonucleic acid, Anti-neutrophil cytoplasmic antibody and Anti-cyclic citrulinated peptide were negative. Magnetic resonance imaging revealed high signal intensity on T2 in both occipital lobes. Skin biopsy of the palm revealed moderate vessel vasculitis. Renal imaging revealed structurally abnormal kidneys with micro aneurysms in the right renal vasculature. Repeat magnetic resonance imaging of the brain two months later showed marked improvement. A diagnosis of polyarteritis nodosa with posterior reversible encephalopathy syndrome was made.

**Conclusions:**

Posterior reversible encephalopathy syndrome should not be missed. Investigations for an aetio-pathological cause should be considered including the rarer associations like polyarteritis nodosa.

## Background

Posterior reversible encephalopathy syndrome (PRES) is a neurotoxic state diagnosed clinico-radiologically. Being introduced in the mid-nineties, it has a classic repertoire of symptoms with alteration of vision, altered mental state, new onset headache, seizures and findings on brain imaging studies with typical magnetic resonance imaging (MRI) and computed tomographic (CT) changes [1]. Even though there is better understanding of its various aetiological causes there is still doubt as to its exact mechanism, and questions remain whether it is hyper-perfusion in the presence of failed auto-regulation with hypertension or endothelial cell dysfunction with resultant vasoconstriction and hypo-perfusion that causes the classic picture [2]. Toxic states in pregnancy, drugs used for immune suppression and chemo therapy, post-transplant, sepsis, and autoimmune conditions have been associated with PRES [3]. Vasculitis is a known aetiology, and is especially seen with a comparative high incidence in systemic lupus erythematosus [4]. However, only a handful of documented cases of polyarteritis nodosa(PAN) with PRES have been reported [5-7]. The mostly benign nature of the disease is demonstrated as there is complete reversal of the clinical picture within days, with symptomatic and aetiologically focused treatment. Principles of treatment include management of hypertension and seizures, and treatment of the causative agent inducing PRES [8]. Reversal of the imaging abnormalities occur anywhere between days to months [1]. Polyarteritis nodosa is a moderate vessel vasculitis that has no definitive diagnostic investigation and the diagnosis is made when clinical criteria are met of standard guidelines such as that of the American College of Rheumatology (ACR) and completed in the presence of either histological evidence or a typical imaging abnormality in the renal arteriogram [9]. We present a typical yet rare case of PAN with PRES.

## Case presentation

A 26-year-old South-Asian female presented with a 6 day history of disproportionately worsened episodic vomiting on a backdrop of a 3 month history of oesophageal reflux due to frequent non steroidal anti- inflammatory drug (NSAID) use, a short two day history of new onset visual impairment with flashes of light, double vision and altered behaviour followed by recurrent generalized tonic-clonic seizures that had an initial focal origin from the left lower limb.

In the preceding year she was investigated for probable pyrexia of unknown origin twice in separate institutions without being able to arrive at a conclusive diagnosis. During the span of one year the patient also noted a weight loss of 8 kilos. She was also treated for eight months for young onset hypertension, which poorly responded to medical management (highest recorded value was 138/110 mmHg, other readings showed fluctuations ranging from 140/90 mmHg to acceptable levels of 110/70 mmHg) even with good drug compliance. She was also diagnosed as having sero-negative arthritis involving the left lower limb knee and ankle and was treated with Non-steroidal anti- inflammatory drugs for the same duration. Three months prior to the presenting episode she developed recurrent left sided loin pain which was diagnosed as a urinary tract infection. For the similar 3 month duration she developed recurrent episodes of vomiting which was diagnosed by upper gastrointestinal endoscopy as antral gastritis secondary to prolonged NSAID use.

**Figure 1:**
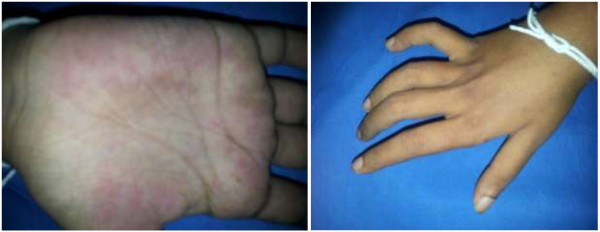
Claw hand and vasculitic rash: Image on the left depicts a palpable vasculitic rash on the palmar surface while the image on the right depicts a right sided claw hand.

On general examination, her consciousness level was a Glasgow coma scale (GCS) level of 14/15 with an elevated blood pressure of 190/120 mmHg. Palms of her hands revealed a blotchy palpable purpuric type of vaculitic rash (Figure 1). Nervous system examination revealed a clawed right hand in isolation without any other deficit or abnormality (Figure 1). Fundi were normal. Remainder of the systemic examination including the cardiovascular and respiratory systems and abdomen was normal.

**Figure 2:**
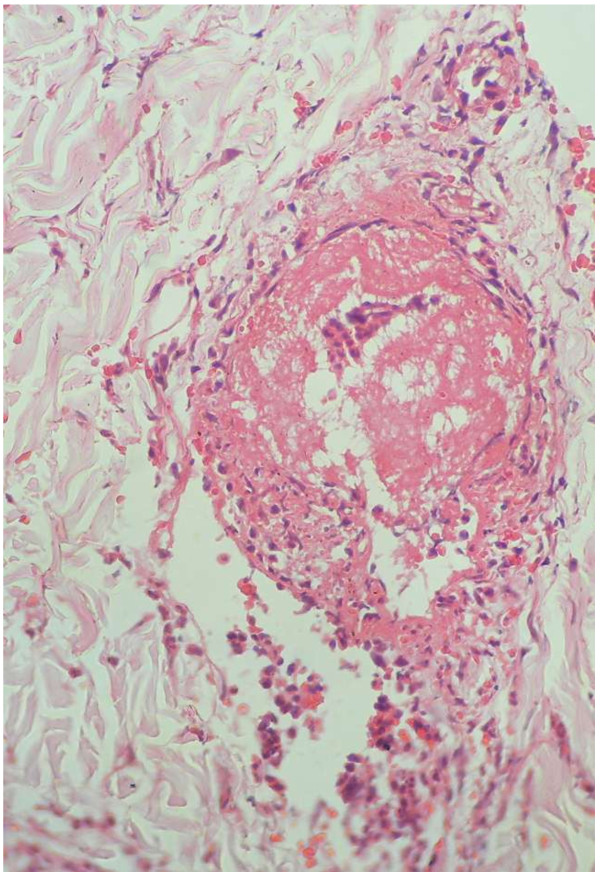
Biopsy findings: Histology demonstrates moderate vessel vasculitis and fibrinoid necrosis with red cell extravasation.

Electrolyte profile was normal, except for serum calcium and phosphate which were ranged in the lower limit of an accepted normal value [S.Ca^2+^- 2.13 mmol/L (2.1-2.55), S.PO_4_^3-^-0.8 mmol/L (0.8-1.5)]. Repeated complete blood counts (CBC) showed markedly elevated white cell count [21.88 × 10^9^/L (4–10)] with neutrophil predominance (85.4 %) and a low haemoglobin value of 7.1 g/dL. All three red cell indices [mean corpuscular volume −70.7 fL (80–100), mean corpuscular haematocrit -21 pg (27–34), mean corpuscular haematocrit concentration- 30 g/dL (32–36)] were reduced. Blood picture demonstrated microcytic hypochromic cells, polychromasia, target cells and polymorphonuclear leukocytosis. Platelet values and morphology were normal. Coombs test was negative. Erythrocyte sedimentation rate was elevated with a value of 130 mm for the 1st hour as was the C – Reactive protein with a value of 114 mg/L. Repeated values too were persistently elevated. Iron studies revealed a reduced serum iron of 8 μg/dL (37–148), a total iron binding capacity of 260 μg/dL (274–385) with an iron saturation of 3.1% (15–50) and an elevated serum ferritin of 189 ng/mL (6–159). Liver functions (coagulation profile and liver enzymes) and renal functions were repeatedly normal. Blood and urine cultures were negative but urinalysis repeatedly showed the presence of a significant number of red cells with protein in the absence of casts. Features were not in favor of meningitis or encephalitis to pursue an investigative path in that direction. Anti-nuclear antibody (ANA) was weakly positive. Anti-double stranded deoxyribonucleic acid (anti-dsDNA), anti-cyclic citrulinated peptide (ACCP), anti-neutrophil cytoplasmic antibodies (ANCA) P and C, cryoglobulin and rheumatoid factor were all negative with an elevated C_3_ complement level of 160 mg/dL with a repeat value of 129 mg/dL (55–120), two weeks later. Thyroid function tests were normal. Hepatitis screening was negative. Skin biopsy revealed moderate vessel vasculitis and red cell extravasation with fibrinoid necrosis of vessels (Figure 2).

**Figure 3:**
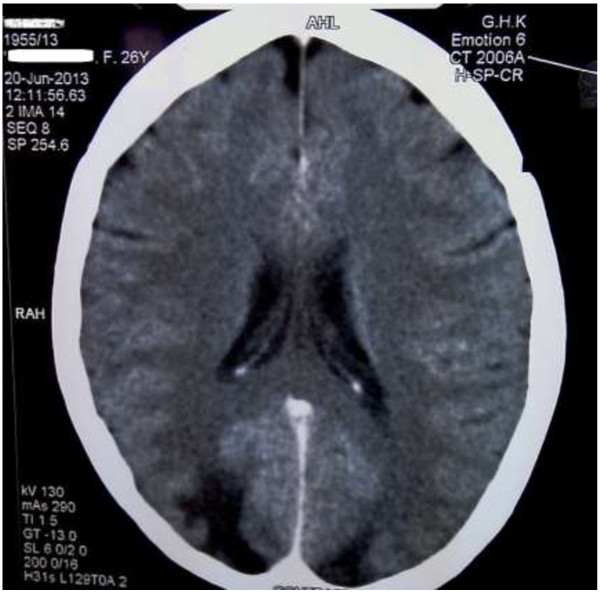
CT brain: Computed tomography scan of the brain taken on preliminary presentation showing bilateral occipital lobe hypodensity and oedema.

**Figure 4:**
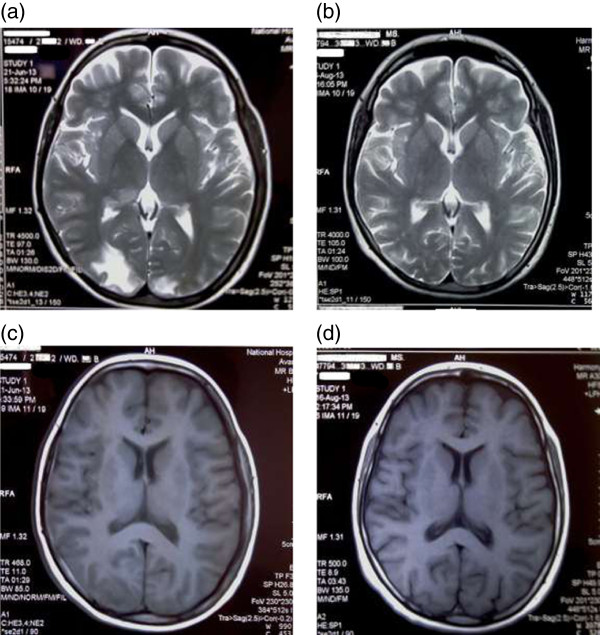
**Preliminary and reassessment MRI brain: (a), (c) are preliminary MRI images taken on presentation. (a)** - T2 weighted shows high signal intensity and **(c)** – T1 weighted shows low signal intensity involving the posterior occipital areas with cortical and subcortical regions. **(b), (d)** are MRI images taken on reassessment showing improvement.

**Figure 5:**
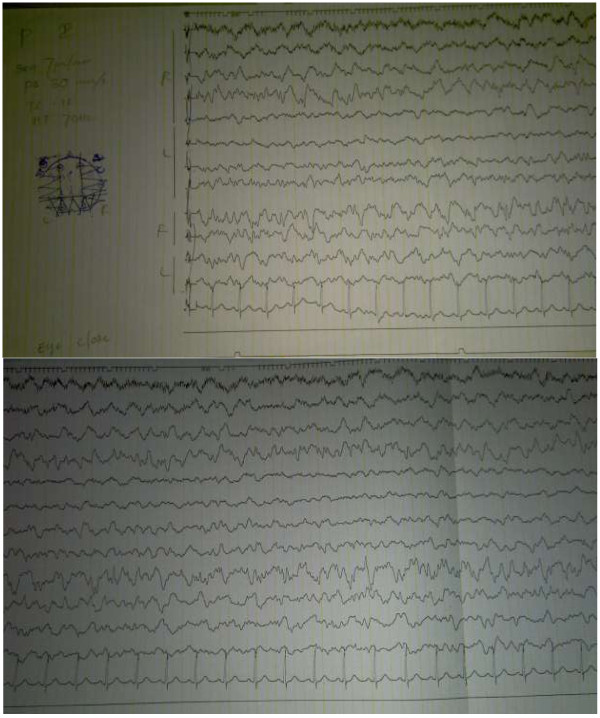
Preliminary EEG on presentation: EEG shows background delta range slowing with continuous right-sided sharp slow complexes suggestive of ongoing seizure discharges.

**Figure 6:**
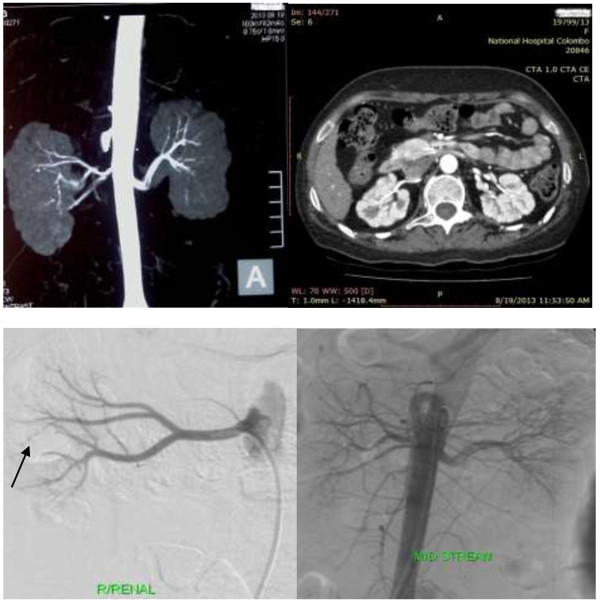
**Renal imaging: Row 1: Computed tomography of the abdomen demonstrates structurally abnormal kidneys with irregular contours, suggesting infarction occurring secondary to PAN causing architectural change.** Row 2: Digital subtraction angiogram of the kidneys demonstrates early aneurysms on the right renal vasculature.

Preliminary non contrast computed tomography of the brain showed bilateral occipital lobe hypodensity coupled with oedema (Figure 3). Magnetic resonance imaging revealed bilateral occipital lobe involvement which had low signal intensity on T1 and high intensity on T2 without gadolinium enhancement (Figure 4). Electroencephalography demonstrated gross background slowing in delta range with continuous right-sided sharp slow complexes suggestive of ongoing seizure discharges, indicating an encephalopathic picture with right focal seizure discharges (Figure 5). Nerve conduction study confirmed isolated ulnar nerve involvement confirming mononeuopathy. Ultrasound abdomen revealed structurally abnormal kidneys (right kidney measured 8.8 cm in length and the left 8.9 cm) with irregular margins but preserved cortico-medullary demarcation. Renal imaging studies confirmed the ultrasound findings (Figure 6). Renal artery angiogram revealed the presence of micro aneurysms in the right kidney vasculature (Figure 6). 2D echo was normal. Two months later the repeat magnetic resonance imaging revealed overall improvement, but with multiple focal high intensity areas being noted on white matter of corona radiata and persistence of a high intensity area on the occipital cortex in the absence of post contrast enhancement (Figure 4).

We treated our patient symptomatically with antiemetics and antihypertensives and controlled the seizures with diazepam satisfactorily. Blood pressure control was achieved and vomiting subsided with a dramatic clinical improvement. Seizures did not persist after the initial day of presentation. Physiotherapy was initiated and continued for the ulnar nerve palsy. Immunosuppressive therapy was initiated for PAN and the patient showed good response with reduction of inflammatory markers and normalization and maintenance of blood pressure.

## Discussion and conclusion

The classic history of seizures, altered behavior and visual disturbance along with brain imaging delineated a diagnosis of PRES. There is variable opinion as to the commonest presenting feature of PRES. Lee *et al.* in their retrospective analysis stated encephalopathy to be the commonest, though Garg considers seizures to precede others and Fugate *et al.* found it to be the commonest (74%) symptom in PRES. However any one of the main features (encephalopathy, seizure, headache, visual disturbance) can be the presenting symptom [10-12]. Speculation exists as to the possible mechanisms resulting in PRES, and there is no clear consensus regarding the exact pathophysiological basis [2,13], and neither do most authors comment on the basis of seizures. But vasogenic oedema, which is seen in both hypo-perfusion and hyper-perfusion could be considered the underlying pathophysiology for the presentations in PRES, including seizures [13,14]. Seizures are commonly generalized and multiple, but can be focal in origin with secondary generalization followed by prolonged altered consciousness, as was in our patient [10,11]. Visual symptoms too are varied e.g., hemianopia, visual neglect, cortical blindness, Anton syndrome, blurred vision [1,10]. Though diplopia is not common it has been reported and can be explained if it involved the brainstem [15]. Imaging usually (94%) reveals symmetrical parieto-occipital lobe involvement, and our patient demonstrated symmetrically distributed occipital lobe changes typically seen on both CT and MRI with associated generalised oedema (Figure 3). Though frequent frontal lobe and to lesser extent cerebellum, brain stem, basal ganglia, deep white matte and even the splenium of the corpus callosum can be involved asymmetrically [8,12]. There may be regional association of presenting symptoms to the area afflicted by PRES, however features such as seizures, headache, encephalopathy make up a spectrum seen in most presentations and seizures are seen even when individual regions (including isolated occipital lobes) were afflicted or when multiple regions are affected together, this was clearly demonstrated in the literature analysis of Leroux *et al.* where presenting symptomology and afflicted radiological areas of involvement of multiple cases of PRES were reviewed [16]. The clinical findings and the history favored a vasculitic picture. Investigational findings (elevated inflammatory markers and low total iron binding capacity with an elevated ferritin) also indicated a chronic process with ongoing inflammation. Imaging studies demonstrated end organ damage involving the kidneys and brain. In the absence of the common autoimmune causes (negative serological findings) and ACR criteria being met for PAN with typical histological finding with renal arterial imaging, a clinical diagnosis of PAN with PRES was made.

Posterior reversible encephalopathy syndrome is a situation that is not very often thought of by clinicians [17]. High level of clinical suspicion is required to include PRES into the differential diagnosis and one may only arrive at the diagnosis following exclusion of commoner acute neurological conditions such as encephalitis, from history and clinical examination. Cerebrospinal fluid analysis may have to be considered if indicated, following initial CT imaging of brain. However CT imaging being the primary investigation of choice, when needed complemented by an MRI provides strong evidence to arrive at a diagnosis of PRES. In our clinical scenario the diagnosis of PRES was made with the typical imaging findings. Angiography studies of the brain vasculature have shown common occurrence of vasculopathy in the form of vasoconstriction, vasodilation and string of bead appearance supporting hypo-perfusion as the cause for PRES with hypertension occurring as a protective counter mechanism [13]. Unfortunately we were unable to do magnetic resonance angiogram of the brain for our patient. Though it would not have changed the final diagnosis or treatment, it would have given further insight into the complexities of the origin of PRES and changes that may have been found in the vessels when hypertension was already present rather than being a protective responsive rise, as the elevated blood pressure being attributable to PAN in our patient, favors the mechanism of resultant auto-regulatory failure and hyper-perfusion induced PRES. In most cases PRES is benign, the hallmark being its reversibility. However permanent neurological injury can occur especially when treatment is delayed [11,18], and in a background of preexistent coagulation abnormalities even intra-cerebral haemorrhage and sub-arachnoid haemorrhage have been reported [19]. In PRES, symptomatic treatment when instituted early results in rapid clinical recovery [17]. When treatment is initiated in the absence of a positive diagnosis, PRES may be missed, especially since neuroimaging changes of this condition have been known to resolve within days [1]. Classically normalization of imaging abnormalities lags behind clinical recovery by weeks or even years [11]. Seizures are usually transient and do not necessitate long term antiepileptic therapy; however rarely chronic epilepsy can occur [11,20].

Since PRES is usually subsequent to a primary aetiopathological process a search for such systemic disease should be initiated. Polyarteritis nodosa by itself is a rare disease. The exclusion of more common autoimmune disease as a possible primary aetiopathological process requires the availability of sero-diagnostic tests. The diagnosis of PAN requires a high degree of suspicion as there is no single investigation that clinches the diagnosis conclusively. In our patient the criteria to suspect PAN was met [9] in the presence of arthralgia, weight loss, mononeuropathy, diastolic blood pressure exceeding 90 mmHg and was strengthened by the skin biopsy evidence of moderate vessel vasculitis and renal arterial imaging demonstrating aneurysms. PAN classically spares the aorta, its tributaries and the lung but can affect any other organs [21]. Renal involvement is seen in about 70% of affected subjects [22]. Our patient demonstrated structurally abnormal kidney architecture. Small persistent infarctions with resultant scarring can alter the renal contour and produce an irregular morphological appearance as was seen in our patient [23]. Digital subtraction angiogram revealed aneurysms of the right renal vessels further signifying that our patient has renal involvement. Other classic vessel abnormalities of medium and small vessels include perfusion defects and delayed emptying of the commonly affected vessels of the kidney e.g., arcuate and interlobular arteries and arterioles [23,24]. PAN can also be staged histologically according to biopsy findings as acute, sub-acute and chronic stages. Since fibrinoid necrosis of vessels is classically seen late and was histologically demonstrated in our patient’s skin biopsy, we could assume that she belonged to the chronic stage of the disease. Central nervous system involvement is also considered a late and less common feature with resultant encephalopathy by Provenzale *et al.*[25]. Biochemically she had iron deficiency anaemia and anaemia of chronic disease, this coupled together with neurological involvement and histology finding suggests long standing disease.

Prior to its new classification as PRES, Rosenberg *et al.* in 1990 have also reported a very similar presenting case of PAN to that of ours [26], possibly implying that cases in the past may have been overlooked and PAN with PRES under-reported. However the situation of PAN resulting in PRES is still uncommon. The atypical presentation of both PAN and PRES, failure to identify each entity, and the lack of availability of sero-diagnostic and imaging studies can result in clinical confusion, delaying early diagnosis and treatment. All of these factors may possibly be why so few cases of PAN and PRES have been reported. In our patient each initial symptom was treated in isolation resulting in drug induced complications while overlooking the overall diagnosis. This reiterates the need for a high level of suspicion for a unifying diagnosis of PAN to avoid isolated symptomatic treatment. This young woman most likely had PAN from the outset causing most of her earlier ailments. The delay in her diagnosis resulting in evolution of the disease to its advanced stage resulting in elevated blood pressure culminated in a presentation of PRES.

## Conclusions

Posterior reversible encephalopathy syndrome requires a high degree of suspicion and should always come into the differential when faced with the classic pattern of symptoms in such an acute presentation. It is a diagnosis which should not be missed and should prompt a thorough search through history, examination and investigations for an aetiopathological cause and consideration should be given even for the less frequent associations. When a vasculitic picture is suggested by the history and examination, even rarer causes should be considered for timely intervention to be instituted.

## Consent

Written informed consent was obtained from the patient for publication of this case report and accompanying images. A copy of the written consent is available for review by the Editor-in-Chief of this journal.

## Abbreviations

PAN: Polyarteritis nodosa; PRES: Posterior reversible encephalopathy syndrome; MRI: Magnetic resonance imaging; CT: Computed tomography; ACR: American College of Rheumatology; ANA: Anti-nuclear antibody; Anti-dsDNA: Anti double stranded DNA; ACCP: Anti-cyclic citrulinated peptide; ANCA: Anti neutrophil cytoplasmic antibodies.

## Competing interests

The authors declare that they have no competing interests.

## Authors’ contributions

TK, CJS, AK, MRN diagnosed the clinical scenario. MRN & AK researched & drafted the documented. All authors provided care for the patient. All authors read and approved the final manuscript.

## Authors’ information

MRN is a registrar of medicine at the National Hospital of Sri Lanka, Colombo.

CJS is a registrar of medicine at the National Hospital of Sri Lanka, Colombo.

AK is a Consultant physician in acute medicine at the National Hospital of Sri Lanka, Colombo.

TK is a senior registrar in medicine at the National Hospital of Sri Lanka, Colombo.
